# Supplementary material for: Validation of KASP-SNP markers in cassava germplasm for marker-assisted selection of increased carotenoid content and dry matter content
Source: Front Plant Sci. 2022 Oct 12;13:1016170. doi: 10.3389/fpls.2022.1016170 (PMC9597466; doi:10.3389/fpls.2022.1016170)
Supplement: Supplementary file 1 [file DataSheet_1.pdf]

Supplementary Material

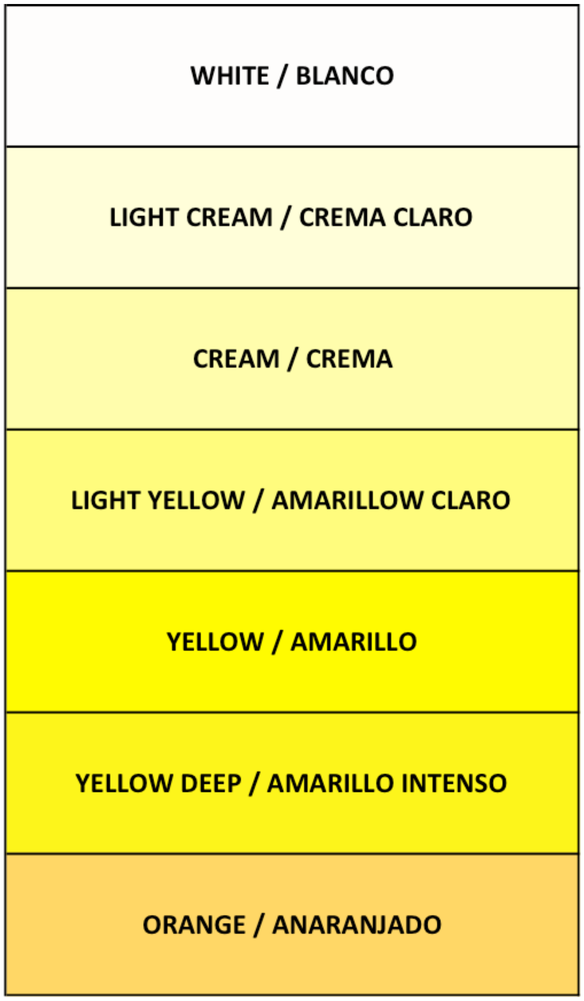

Supplementary Figure 1: Standard color-chart for scoring cassava tissue for carotenoid content

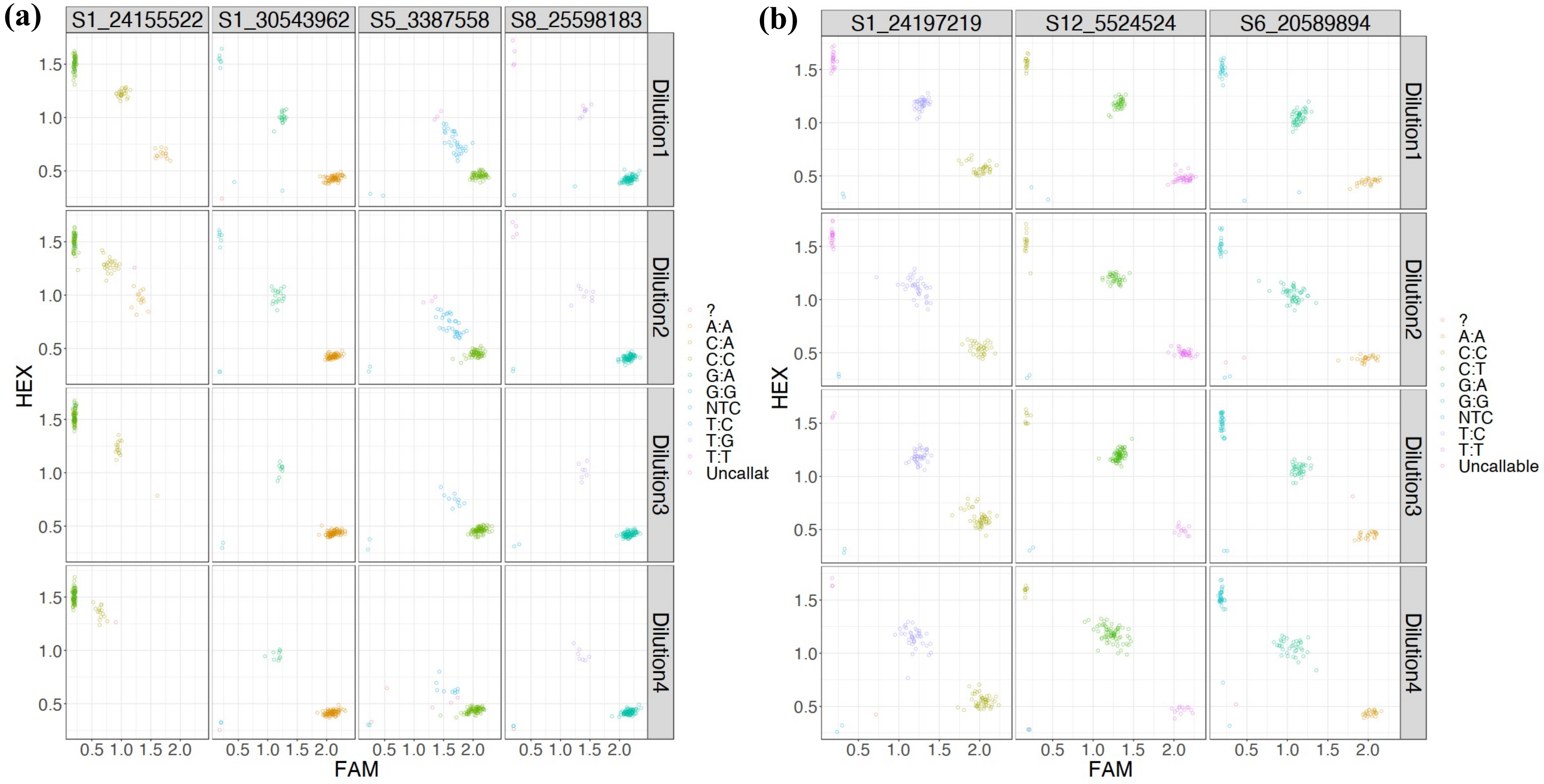

Supplementary Figure 2: Scatter plots of genotypic classes from the KASP marker assays associated with increased (a) carotenoid content and (b) dry matter content in the 188 diverse cassava germplasm used for the technical validation at four DNA concentrations (Dilution 1= 10X, Dilution 2 = 100X, Dilution 3 = 24X, Dilution 4 = 240)

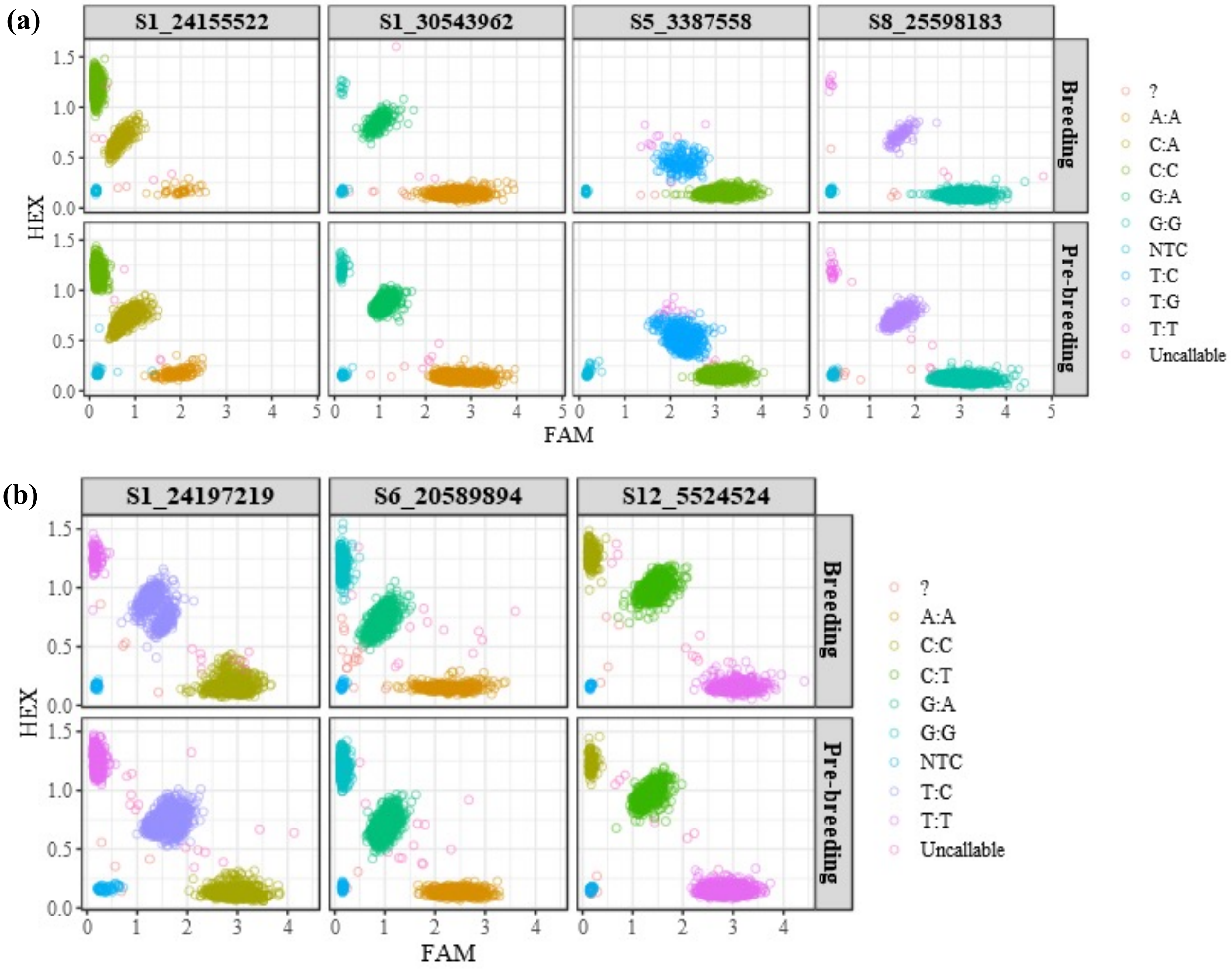

Supplementary Figure 3: Scatter plots of genotypic classes from the KASP marker assays associated with increased (a) carotenoid content and (b) dry matter content in the breeding and pre-breeding populations

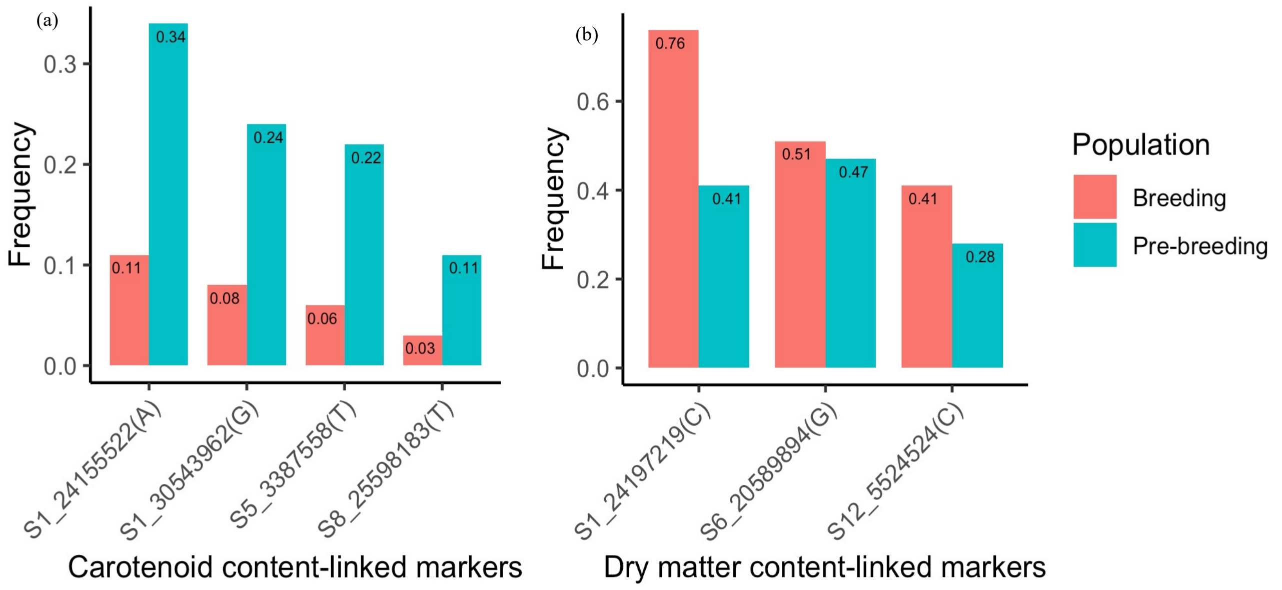

Supplementary Figure 4: Frequencies of favorable alleles at the markers associated with increased (a) carotenoid and (b) dry matter content in the breeding and pre-breeding populations

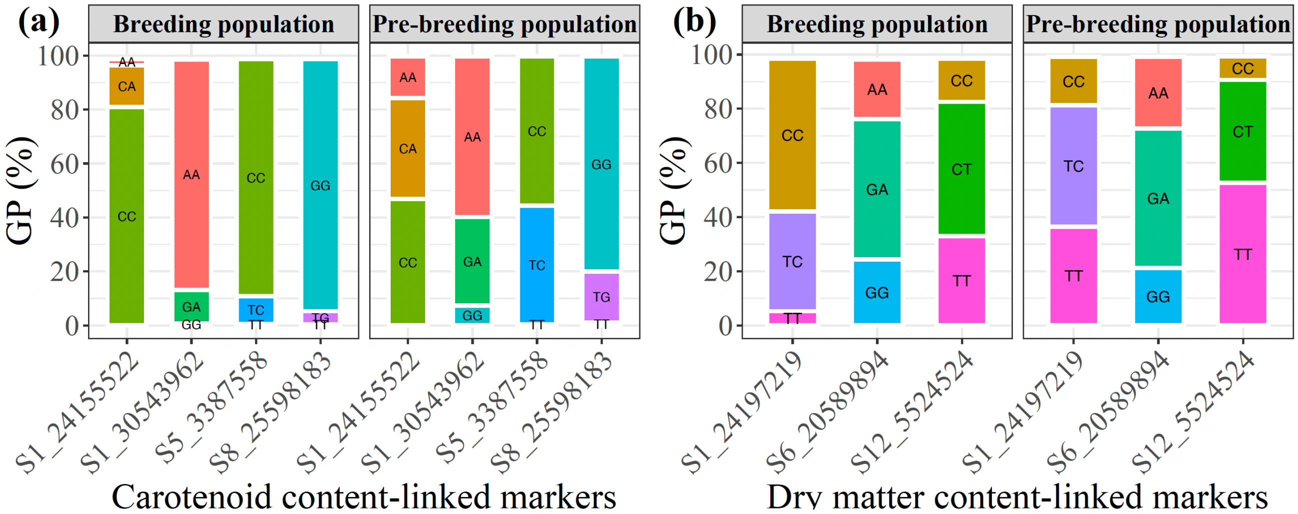

Supplementary Figure 5: Marker genotype frequencies associated with increased (a) carotenoid content and (b) dry matter content in the breeding and pre-breeding populations GP: Genotype percentage

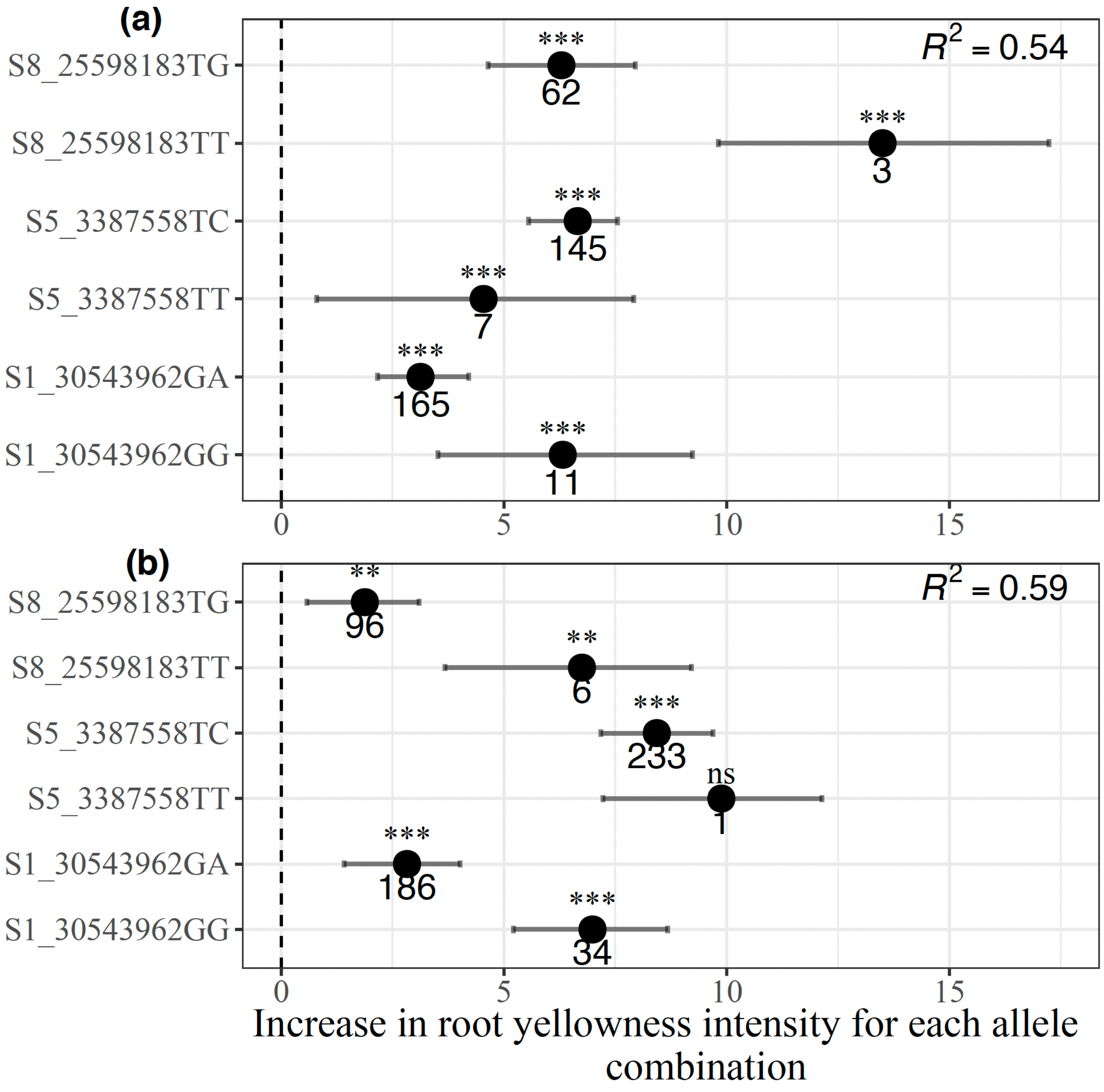

Supplementary Figure 6: Distribution of marker allelic effects associated with increased carotenoid content in (a) breeding, and (b) pre-breeding populations (The multiple linear regression model excluded marker S1_24155522)

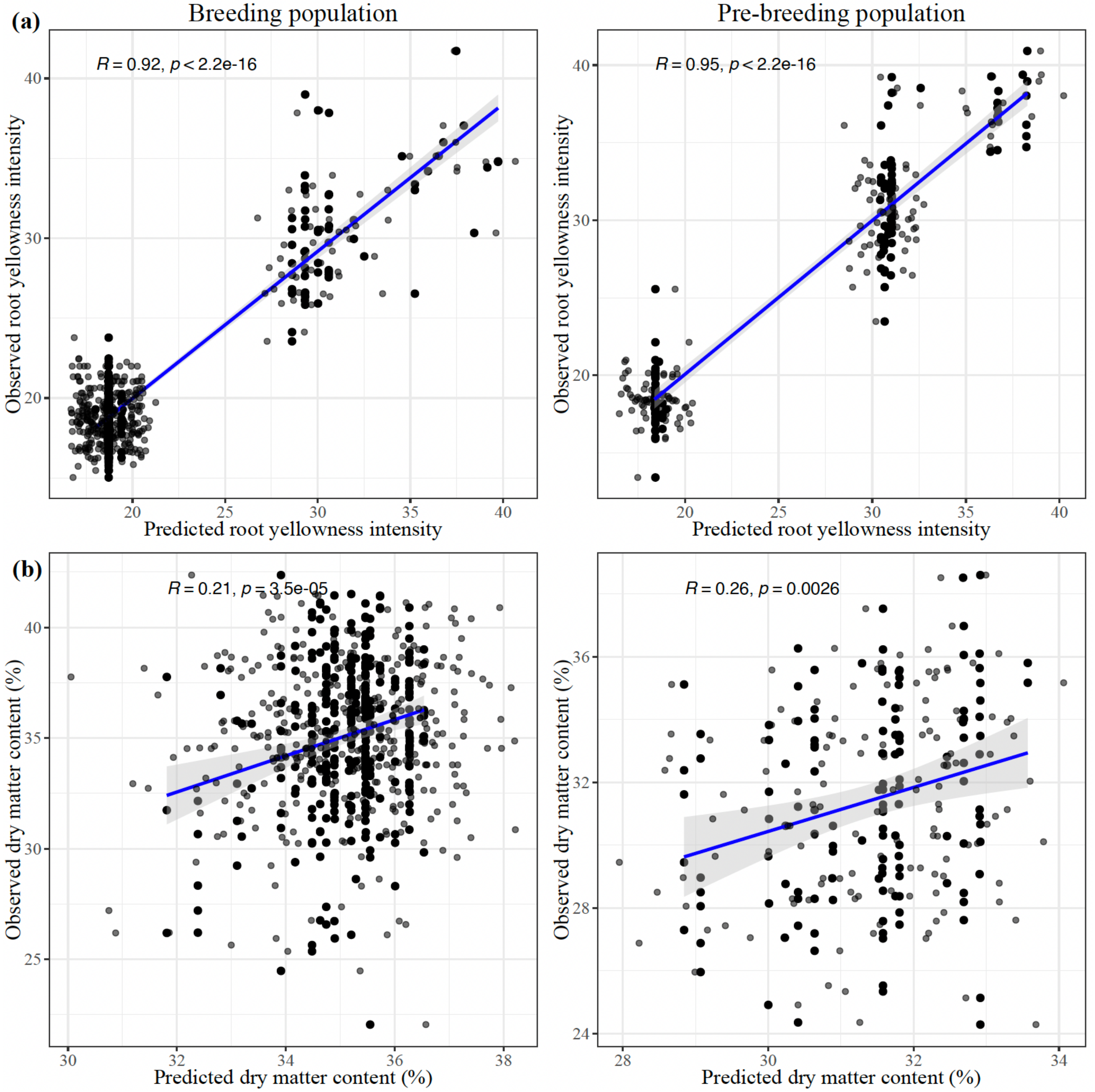

Supplementary Figure 7: Predictive accuracies of the markers associated with increased (a) carotenoid content and, (b) dry matter content in the testing sets of the breeding and pre-breeding populations

Supplementary Table 1: Number of predicted genotypes and uncallable for markers linked to increased carotenoid content and dry matter content in the breeding and pre-breeding populations

**Traits**	**Populations**	**S1_24155522**				
		**AA**	**CA**	**CC**	**Uncallable**	**Call rate (%)**
	Breeding	40	271	1416	23	98.7
	Pre-breeding	122	296	370	2	99.7
		**S1_30543962**				
		**GG**	**GA**	**AA**	**Uncallable**	**Call rate (%)**
	Breeding	14	216	1495	25	98.6
	Pre-breeding	58	259	470	3	99.6
		**S5_3387558**				
		**TT**	**TC**	**CC**	**Uncallable**	**Call rate (%)**
	Breeding	9	181	1537	23	98.7
	Pre-breeding	3	348	437	2	99.7
		**S8_25598183**				
		**TT**	**TG**	**GG**	**Uncallable**	**Call rate (%)**
	Breeding	7	85	1635	23	98.7
Carotenoid content	Pre-breeding	8	150	629	3	99.6
		**S1_24197219**				
		**CC**	**TC**	**TT**	**Uncallable**	**Call rate (%)**
	Breeding	988	644	92	26	98.5
	Pre-breeding	141	354	288	7	99.1
		**S6_20589894**				
		**GG**	**GA**	**AA**	**Uncallable**	**Call rate (%)**
	Breeding	427	905	385	33	98.1
	Pre-breeding	168	406	209	7	99.1
		**S12_5524524**				
		**TT**	**CT**	**CC**	**Uncallable**	**Call rate (%)**
	Breeding	577	867	280	26	98.5
Dry matter content	Pre-breeding	416	300	69	5	99.4
